# Cultivating courage: Interview with an established leader in an innovative practice

**DOI:** 10.1177/17151635231215064

**Published:** 2023-12-03

**Authors:** Liam Jackman

**Affiliations:** Institute of Health Policy, Management and Evaluation, University of Toronto, Toronto, Ontario; Temerty Faculty of Medicine, University of Toronto, Toronto, Ontario

Rahim Dhalla is a pioneer in providing patients with access to medical cannabis, an emerging area in health care with the potential to help many.^1^ He is the owner and operator of Hybrid Pharm, Canada’s only pharmacy specializing in medical cannabis, located in Ottawa, Ontario. I interviewed Dr. Dhalla because of his innovative spirit and success leading a ground-breaking practice. The interview encouraged me to think about leadership, the skills that contribute to its success and how these may apply to those interested in becoming effective health care leaders. Ultimately, leadership is about inspiring a shared vision and taking decisive action to bring it to reality. Dr. Dhalla asserts that courage, the ability to confront uncertainty,^2^ is the most important leadership skill, as it is necessary to effectively execute decision-making in uncertain situations.

Courage, like a muscle, can be trained and strengthened.^3^ There are different types of courage, as there are different groups of muscles, and these are activated at specific times to perform specific functions. Treasurer,^4^ author of *Courage Goes to Work: How to Build Backbones, Boost Performance, and Get Results*, presents a comprehensive conceptualization of courage, dividing it into 3 fundamental categories: try, trust and tell courage. *Try courage* is the courage of action, *trust courage* is the courage of relying on the actions of others and *tell courage* is the courage of voice.

The first type of courage, *try courage*, is essential to effective leadership, as it drives change.^4^ It takes courage to try something new because in doing so, one assumes risk. There is a risk of failure, which can harm oneself and others.^5^ This risk causes most people to recede in their attempt to take action, but an effective leader assumes the brunt of this risk and persists in their attempt to do so. This contributes to a culture of continuous improvement where teams are able to adapt to changing health care landscapes and address complex health care challenges by trying something new.^6^ The secret to transforming the Canadian Health Care System, according to Dr. Dhalla, is having the courage to try to apply existing knowledge in new ways. He demonstrates try courage by advocating for access to medical cannabis despite the stigma surrounding its use. He persists in his attempts to learn more about its uses, particularly the benefits and risks, and in doing so has advanced care options and outcomes for patients. Ultimately, he has become a pioneer in the field and a trusted source of up-to-date information for patients and providers alike. To cultivate try courage, emerging leaders can open up to opportunities to try something new and, in turn, increase their risk threshold.^2^ They can practise thinking through scenarios, considering the risks of taking action and, sometimes more importantly, the risks of inaction.^7^ The support of a seasoned mentor known for their willingness to embrace change can also facilitate this process.

**Figure:**
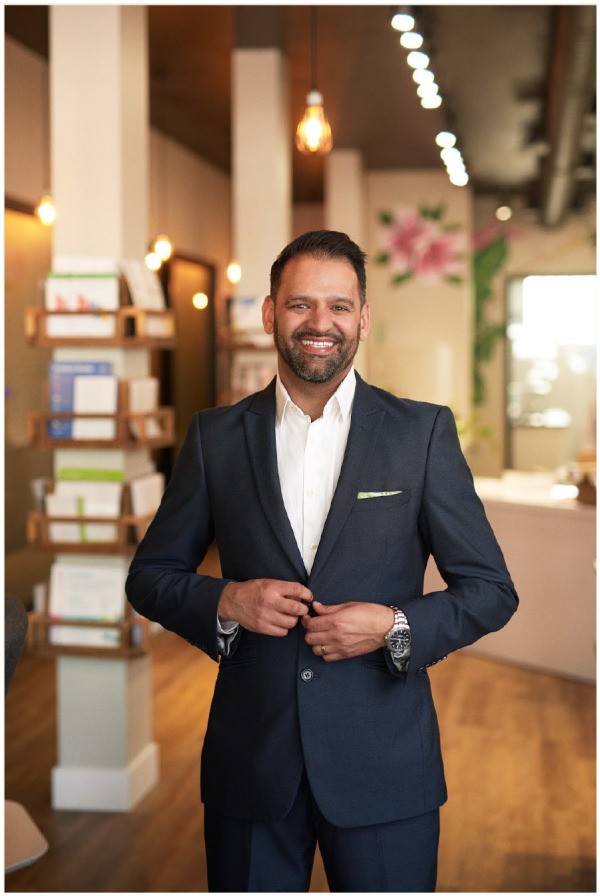
Rahim Dhalla

The second type of courage, *trust courage*, is essential to effective leadership, as it builds bonds and encourages employee engagement through the promotion of psychological safety.^8^ It takes courage to trust others because in doing so, one cedes control and becomes vulnerable to the consequences of another’s actions.^9^ However, an effective leader leans into this discomfort and delegates to others. This contributes to a culture of learning, where team members challenge themselves to learn more about the scope of a problem and propose their own solutions. This way, team members are empowered to expand on their knowledge and become more engaged in their work, as their contributions are seen as being of value and worthy of trust.^8^ The secret to leadership success, which Dr. Dhalla wishes he had learned sooner, is to lean on one’s network. He demonstrates trust courage by conducting cannabis consultations in which he is transparent about the scope of his knowledge, including its limits. He empowers his team to conduct cannabis consultations on their own, encouraging the same level of transparency. This transparency creates a culture of psychological safety, as team members trust each other enough to share uncertainties and see them as learning opportunities rather than failures. To cultivate trust courage, emerging leaders can participate in workshops on emotional intelligence and participate in team-building exercises to enhance trust-building skills.^8^ They should practise leading by example and be open to vulnerability, as doing so creates supportive environments where everyone is heard and respected, something instrumental in nurturing trust courage.^10^

The third type of courage, *tell courage*, is essential to effective leadership, as it aligns team members to a shared vision. It takes courage to share one’s voice, particularly when it runs contrary to that of the group, as there is a risk of being cast out of the group.^4^ However, an effective leader speaks up and asserts themselves when they feel strongly about something. The leader who demonstrates tell courage inspires their team members to do the same, contributing to a culture where the status quo is challenged rather than simply accepted and even amplified.^11^ Dr. Dhalla demonstrates tell courage by speaking up against the sale and distribution of accessories for combustible cannabis products in medical cannabis spaces. He faces pressures from business and industry partners to participate but asserts that to do so is against his vision of improving patient health. Through tell courage, he upholds his integrity and encourages others to do the same.^11^ To cultivate tell courage, emerging leaders can engage in role-playing exercises that simulate challenging conversations. These serve as opportunities to prepare their thoughts and practise their delivery.^12^ There are also workshops on communication that can be undertaken to further enhance assertiveness.^13^

In conclusion, courage is an essential skill for the emerging health care leader, as it is through the development and demonstration of try, trust and tell courage that they equip themselves and their teams to confront uncertainty in their pursuit of positive change. ■
